# Hedonic Value and Normative Goal: The Role of Interdependent Self-Construal in the Evaluation of Plant-Based Milk Alternatives

**DOI:** 10.3390/foods15213864

**Published:** 2026-10-28

**Authors:** Jin-Hong Park, Minguk Seo, Lana Chung

**Affiliations:** 1 Department of Culinary Science and Food Service Management, Graduate School, Kyung Hee University, 26 Kyungheedae-ro, Dongdaemun-gu, Seoul 02447, Republic of Korea; doorgoor120@naver.com (J.-H.P.); culinary_edu@naver.com (M.S.); 2 Department of Culinary Arts & Food Design Management, College of Hotel & Tourism Management, Kyung Hee University, 26 Kyungheedae-ro, Dongdaemun-gu, Seoul 02447, Republic of Korea

**Keywords:** plant-based milk alternatives, Goal-Framing Theory, hedonic value, biospheric value, normative goal, interdependent self-construal

## Abstract

Plant-based milk alternatives (PBMAs) have gained attention as part of sustainable dietary transitions, yet consumer evaluations may reflect both environmental and hedonic considerations. Hedonic value was of particular theoretical interest because its relationship with normative goals is less theoretically established than that of biospheric value. Drawing on Goal-Framing Theory (GFT), this study examined how biospheric and hedonic values are associated with normative goals, whether normative goals are associated with attitude toward PBMAs, and whether interdependent self-construal moderates the association between hedonic value and normative goal. A cross-sectional online survey was conducted through a Korean research panel with a final analytic sample of 288 consumers aged 20–39 who had prior experience consuming plant-based beverages. All respondents received the same environmental information before completing the study measures. Confirmatory factor analysis and PROCESS-based indirect-effect and moderation analyses were conducted. Biospheric and hedonic values were positively associated with normative goals, which were positively associated with attitude toward PBMAs. Both values showed significant indirect associations with attitude through normative goals. The association between hedonic values and normative goals was not statistically significant at a low level of interdependent self-construal but was positive at the mean and high levels. These findings suggest that a general hedonic value orientation is not necessarily incompatible with normative goals in the environmental-information context examined in this study and that interdependent self-construal may represent a relevant boundary condition in value–goal associations.

## 1. Introduction

Food production and consumption impose substantial environmental burdens, including greenhouse gas emissions and extensive land and resource use. Accordingly, the transition toward more sustainable food systems requires not only changes in production practices but also shifts in consumer food choices [1]. Plant-based alternatives have consequently received growing attention as part of more sustainable dietary transitions. Plant-based milk alternatives (PBMAs) represent a prominent product category within this shift, yet their acceptance depends on more than environmental considerations. Sensory and non-sensory factors, including taste, texture, familiarity, and consumption context, also shape consumer responses to plant-based foods and beverages [2,3,4]. Recent research on plant-based milks likewise indicates that sensory attributes and consumption motivations remain important to consumer liking and product use [5]. PBMA evaluation may therefore involve environmental and hedonic considerations simultaneously.

Research on plant-based food acceptance has identified multiple determinants, including environmental and health perceptions, sensory attributes, familiarity, attitudes, and social norms [6,7]. Existing accounts of sustainable consumption have also emphasized relatively stable values, environmental self-identity, environmental beliefs, personal norms, and moral goals [8,9,10]. These constructs are conceptually distinct from the situationally focal motivational goals emphasized in Goal-Framing Theory (GFT). Values represent relatively stable orientations, environmental self-identity reflects how environmental concern is incorporated into the self-concept, and personal or moral norms concern perceived obligations; in contrast, a normative goal in GFT represents a focal motivational orientation that directs attention toward what is appropriate to do in a particular situation. Examining normative goals may therefore provide a more proximal motivational link between relatively stable value orientations and context-specific product evaluations.

This distinction is particularly relevant to PBMAs because consumers may simultaneously value environmental protection and seek pleasure and satisfaction from food consumption. Whereas biospheric value has a relatively clear theoretical affinity with normative goal, the relationship between hedonic value and normative goal is less theoretically established. This asymmetry makes hedonic value a particularly relevant focal construct for examining whether pleasure-oriented values can coexist with normative motivation in sustainable food evaluation. Examining biospheric and hedonic value orientations within the same motivational framework may therefore provide a more integrated account of PBMA evaluation.

Goal-Framing Theory (GFT) provides a useful framework for examining these relationships. GFT distinguishes three overarching goal frames—normative, hedonic, and gain—and proposes that multiple goals may be active simultaneously, while their relative strength can vary according to individual values and situational conditions [11,12]. Biospheric value emphasizes concern for nature and environmental protection and is theoretically compatible with a normative orientation, whereas hedonic value represents a distinct orientation toward pleasure and comfort [13]. A key conceptual distinction is that hedonic value is not equivalent to a hedonic goal frame. Values represent relatively stable orientations, whereas the relative strength of goal frames may vary across situations. GFT allows different goals to conflict or coexist depending on contextual conditions [11,14]. Research in food settings similarly indicates that goal salience and goal conflict can vary across contexts [15,16]. More recent applications have continued to apply GFT to sustainability-related decisions, including recycling and sustainable food choice [9,17]. However, these studies do not directly examine how a relatively stable hedonic value orientation is related to normative goals in the specific context of PBMA evaluation. Thus, this value–goal relationship remains insufficiently understood.

The value–goal relationship may also depend on how individuals construe the self. Interdependent self-construal refers to a tendency to understand the self in relation to others and to place greater emphasis on connectedness, relational harmony, and social context [18,19]. Evidence from pro-environmental and green-consumption research suggests that self-construal can condition responses to social norms [20,21]. Although social norms are conceptually distinct from normative goals in GFT, these findings provide a basis for examining whether interdependent self-construal also conditions norm-related motivational processes within a GFT framework. Whether it conditions the relationship between hedonic value and normative goal, however, remains largely unexplored.

Accordingly, this study examines how biospheric and hedonic values are associated with normative goals and how normative goals are associated with attitudes toward PBMAs among Korean consumers, within a common evaluation context in which all respondents received the same environmental information. It also examines the indirect associations between the two value orientations and attitude toward PBMAs through normative goals, as well as whether interdependent self-construal moderates the relationship between hedonic value and normative goal. This study makes three theoretical contributions. First, it distinguishes the theoretically expected relationship between biospheric value and normative goal from the less established relationship between hedonic value and normative goal, thereby clarifying why hedonic value constitutes a focal theoretical issue in the present study. Second, it examines normative goals as a proximal motivational pathway through which relatively stable value orientations are associated with attitude toward PBMAs, thereby complementing explanations centered on values, environmental self-identity, or personal norms. Third, it examines interdependent self-construal as a potential boundary condition of the hedonic value–normative goal relationship, extending the application of GFT by considering individual differences in how value orientations are associated with context-specific motivational goals.

## 2. Theoretical Framework and Development of Hypotheses and Research Questions

Goal-Framing Theory (GFT) proposes that multiple goals can be active simultaneously, while the relatively strongest, or focal, goal frames individuals’ attention, information processing, judgment, and behavior [11]. GFT distinguishes three overarching goal frames: normative, hedonic, and gain goals. In environmental decision-making, the relative strength of these goals may depend on individual value orientations as well as situational factors [12]. Building on this framework, the following sections develop the theoretically expected relationship between biospheric value and normative goal, examine the less established relationship between hedonic value and normative goal, develop the hypothesized association between normative goal and attitude toward PBMAs, consider indirect associations through normative goal, and examine interdependent self-construal as a potential boundary condition of the hedonic value–normative goal relationship.

### 2.1. Biospheric Value and Normative Goal

Biospheric value refers to a relatively stable value orientation that emphasizes the protection of nature and the environment [22,23]. It is empirically distinguishable from egoistic and altruistic value orientations and is closely related to environmental beliefs and intentions [23]. Within GFT, the normative goal directs attention toward what is considered right and appropriate in a given situation [11]. Its relative strength may depend on individual values and situational factors, with biospheric value being particularly relevant to normative goals in environmental decision-making [12]. Accordingly, individuals who place greater importance on biospheric value are expected to report a stronger normative goal in an environmental evaluation context.

Related value–norm processes have also been observed in plant-based food consumption. Wang and Chang [10] found that biospheric values were positively associated with environmental beliefs, which were in turn positively related to personal norms regarding plant-based foods. Although personal norm is conceptually distinct from the normative goal specified in GFT, this finding provides adjacent evidence that biospheric values are linked to norm-related psychological processes in plant-based food choice. The relationship between biospheric value and GFT-based normative goal has nevertheless received limited direct examination in the PBMA context. Compared with hedonic value, however, biospheric value has a clearer theoretical affinity with normative goals because concern for environmental protection is consistent with a normative orientation toward acting appropriately in an environmental context. Thus, the biospheric value–normative goal relationship represents a theoretically expected relationship, whereas the corresponding relationship between hedonic value and normative goal is less established. Accordingly, the following hypothesis is proposed:

**H1.**  *Biospheric value is positively related to normative goals.*

### 2.2. Hedonic Values and Normative Goals

By contrast with biospheric value, hedonic value reflects a relatively stable value orientation that emphasizes pleasure, enjoyment, and personal gratification and is empirically distinct from egoistic, altruistic, and biospheric values [13]. Importantly, hedonic value in the present study refers to a general orientation toward enjoyment and gratification in life rather than to product-specific sensory pleasure or the perceived taste of PBMAs. Previous research has shown that hedonic value can be negatively associated with environmentally relevant attitudes, preferences, and behaviors [13]. However, these findings concern environmental outcomes more broadly and do not establish how a relatively stable hedonic value orientation is related to normative goals.

Within GFT, hedonic value and a hedonic goal frame are conceptually distinct. Values represent relatively stable orientations, whereas goal frames refer to situationally focal motivational orientations whose relative strength may vary according to both individual values and contextual conditions [11,12,14]. Because multiple goals may be active simultaneously, hedonic and normative considerations are not necessarily mutually exclusive. Accordingly, a strong hedonic value orientation does not necessarily imply a weaker normative goal. This distinction makes the relationship between hedonic value and normative goal theoretically less straightforward than the relationship between biospheric value and normative goal.

Evidence from sustainable food contexts further supports the context-dependent nature of goal-related processes. Erhard et al. [16] found that misalignment between an active hedonic goal and sustainability-oriented attribute framing reduced taste expectations and engagement with plant-based alternatives, while Onwezen [15] showed that the salience and relevance of different goal frames can vary across food-choice contexts. Recent applications of GFT have further demonstrated the relevance of situational and contextual conditions to goal-related processes in sustainability-related behavior and sustainable food choice [9,17]. However, these studies primarily concern situationally activated goals or goal-framing processes rather than the relationship between a relatively stable hedonic value orientation and normative goals. Thus, despite recent developments in GFT research, direct evidence regarding the direction of the hedonic value–normative goal relationship remains limited, particularly in the context of PBMA evaluation. Therefore, unlike the theoretically expected positive relationship between biospheric value and normative goal proposed in H1, no directional relationship is specified a priori for hedonic value. The following research question is proposed:

**RQ1.**  *How is hedonic value related to normative goals?*

### 2.3. Normative Goal and Attitude Toward PBMAs

According to GFT, goal frames shape how individuals attend to information, evaluate alternatives, and form judgments in a given situation [11]. When a normative goal is relatively strong, individuals are more likely to evaluate available options in terms of what is considered appropriate or ought to be done and to give greater weight to attributes that are consistent with this orientation. Accordingly, within the shared environmental-information context of the present study, a stronger normative goal is expected to be associated with a more favorable attitude toward PBMAs.

Related research on norm-based motivation provides adjacent, rather than direct, support for this expectation. Personal norms, moral goals, social norms, and the GFT-based normative goal are conceptually distinct constructs, although each concerns norm-related processes relevant to sustainable consumption. Khan et al. [24], for example, found that normative motivation was positively associated with purchase intention for organic food. Onwezen [15] further demonstrated the relevance of normative and moral goal frames across sustainable food-choice contexts, while more recent GFT research has continued to examine goal-related processes in sustainable food choice [9]. However, these studies do not directly establish the relationship between the GFT-based normative goal and attitude toward PBMAs. Moreover, previous research has often focused on behavioral intentions or other consumption responses rather than product-specific attitude. Thus, the association between normative goal and attitude toward PBMAs remains insufficiently examined. Accordingly, the following hypothesis is proposed:

**H2.**  *Normative goals are positively related to attitude toward PBMAs.*

### 2.4. Indirect Role of Normative Goals

Within GFT, values represent relatively stable orientations, whereas goal frames more proximally organize attention and evaluation in specific situations [11,14]. Normative goals may therefore represent a proximal motivational pathway through which value orientations are associated with context-specific product evaluations.

Related research provides adjacent support for this general value–proximal psychological process–response pattern. van der Werff et al. [8] showed that biospheric values were associated with environmental preferences, intentions, and behavior through environmental self-identity. In the plant-based food context, Wang and Chang [10] similarly found that biospheric values were associated with environmental beliefs, which in turn were related to personal norms and repurchase intentions. Although environmental self-identity and personal norms are conceptually distinct from the GFT-based normative goal, these findings suggest that relatively stable values may be associated with consumer responses through more proximal psychological processes.

Based on this reasoning, an indirect association between biospheric value and attitude toward PBMAs through normative goals is expected. Although the direction of the hedonic value–normative goal relationship is not specified a priori (RQ1), the value–goal logic of GFT also provides a basis for expecting an indirect association between hedonic value and attitude toward PBMAs through normative goal. Accordingly, the following hypotheses are proposed:

**H3.**  *Biospheric value is indirectly related to attitude toward PBMAs through normative goals.*

**H4.**  *Hedonic value is indirectly related to attitude toward PBMAs through normative goals.*

### 2.5. Theoretical Rationale for the Moderating Role of Interdependent Self-Construal

Interdependent self-construal refers to the tendency to understand the self in relation to others and to emphasize connectedness, relational harmony, and social context [18,19]. Individuals higher in interdependent self-construal may therefore be more attentive to socially relevant cues and relational considerations when evaluating appropriate courses of action.

Research on sustainable consumption provides adjacent evidence for this possibility. Saracevic and Schlegelmilch [20], in their systematic review, identified culture and self-construal as important contingencies in the relationship between social norms and pro-environmental behavior. More specifically, Hu and Hu [21] found that descriptive norms were more effective in activating impression management among consumers higher in interdependent self-construal, with subsequent associations with green consumption attitudes and behavior. Although social norms and the normative goal in GFT are conceptually distinct, these findings suggest that interdependent self-construal may be relevant to individual differences in norm-related motivational processes.

The present study focuses specifically on the hedonic value–normative goal relationship because, unlike the theoretically more congruent biospheric value–normative goal relationship, the association between hedonic value and normative goal is less theoretically established. As discussed in Section 2.2, hedonic value reflects a general orientation toward enjoyment and gratification, whereas normative goal concerns what is considered appropriate to do in a particular context. The extent to which these distinct orientations are associated may therefore vary according to individual differences in relational self-orientation. Interdependent self-construal may represent one such boundary condition because it reflects the degree to which individuals define themselves in relation to others and attend to relational and social considerations. Accordingly, the relationship between hedonic value and normative goal may vary across levels of interdependent self-construal.

Because direct evidence regarding this specific moderating relationship remains limited, and its direction cannot be specified confidently a priori, the following research question is proposed:

**RQ2.**  *Does interdependent self-construal moderate the relationship between hedonic value and normative goal?*

**Figure 1:**
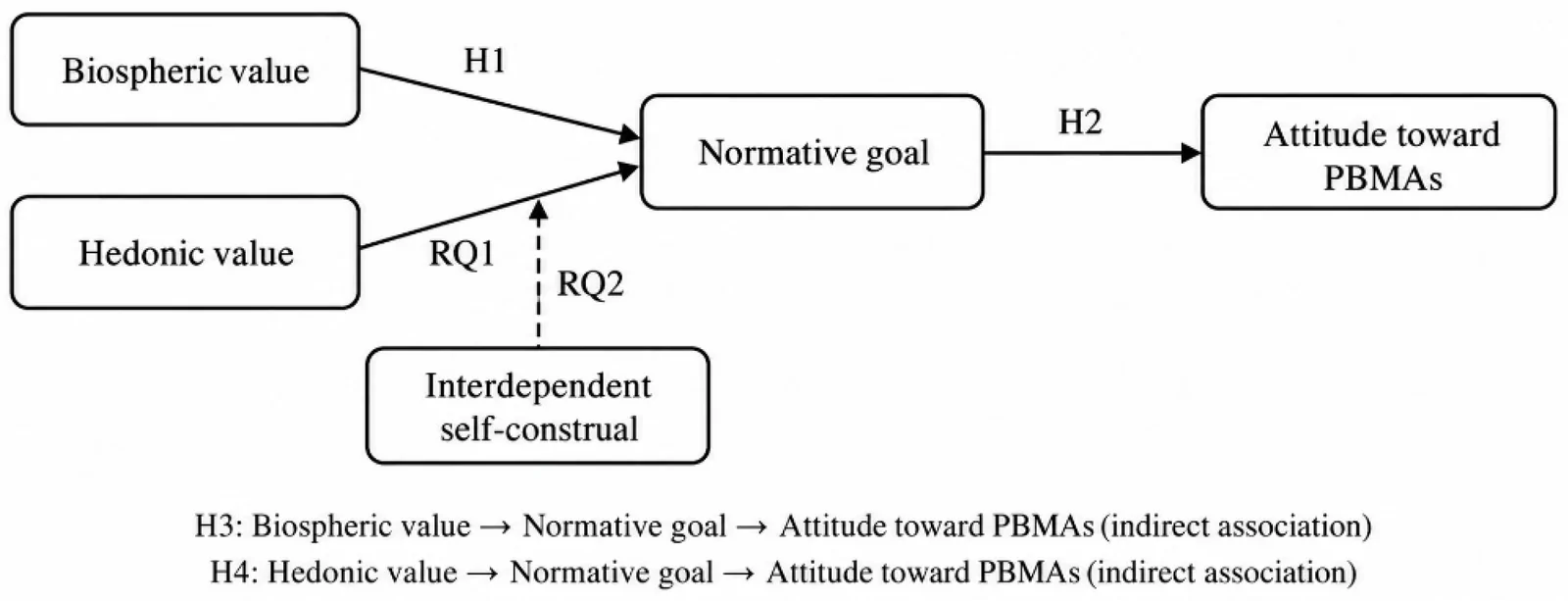
Research model.

The proposed research model is presented in Figure 1.

## 3. Materials and Methods

### 3.1. Participants and Data Collection

This study employed a cross-sectional online survey of consumers aged 20–39 years in South Korea. The study focused on this age group because previous research on plant-based milk consumption in Korea found that consumers in their 20s and 30s were relatively more highly represented among consumers of non-soy plant-based milk alternatives than among soy-milk consumers [25]. Accordingly, the present study focused on younger adult consumers as a relevant segment of the Korean PBMA market rather than seeking to represent the entire Korean adult population. Participants were recruited through Embrain, a Korean online research panel, and data were collected from 1 September to 9 September 2025.

Eligibility was determined using a screening question assessing prior consumption experience. Respondents were asked to select all beverage categories they had previously consumed, including plant-based beverages such as soy milk, almond milk, and oat milk. For the purposes of this study, PBMAs were operationalized as plant-based beverages used as alternatives to dairy milk, including soy milk, almond milk, and oat milk. Only respondents who selected the plant-based beverage category were allowed to proceed with the survey. Thus, eligibility was based on prior consumption experience rather than current consumption frequency.

A total of 350 eligible respondents completed the survey. A post hoc data-quality screening procedure was conducted to identify potentially inattentive, inconsistent, or incomplete responses. Initially, 47 responses were excluded through manual screening, including 10 exhibiting repetitive zigzag patterns, 22 showing straight-lining, and 15 containing missing data. Subsequently, construct-level mean scores were calculated, and box-plot-based outlier screening resulted in the exclusion of an additional 15 responses. The final analytical sample comprised 288 respondents. Because the exclusion criteria were not prospectively specified, the post hoc nature of the screening procedure should be considered when interpreting the findings.

PBMA consumption frequency was assessed separately after eligibility screening. The consumption-frequency item did not specify an explicit retrospective reference period. Among the 288 respondents included in the final analytic sample, 41 selected “Never” on the consumption-frequency item. These respondents were retained in the primary analyses because they had previously reported consumption of plant-based beverages during eligibility screening. To assess whether their inclusion affected the findings, the indirect-effect, moderation, and conditional indirect-effect analyses were repeated after excluding these 41 respondents (N = 247). The substantive findings remained statistically consistent in these sensitivity analyses (Supplementary Section S4, Table S6).

Before participation, all respondents were informed of the purpose and procedures of the study and the voluntary nature of participation, and informed consent was obtained from all participants. Participation was anonymous, and no personally identifiable information was collected. The characteristics of the final analytic sample are presented in Table 1.

### 3.2. Survey Procedure

To provide a common context for evaluating plant-based milk alternatives (PBMAs), all respondents were presented with the same visual information comparing the environmental impacts of PBMAs and dairy milk. The information addressed global warming potential, acidification potential, eutrophication potential, and land use and was developed based on the life-cycle evidence reviewed by Khanpit et al. [26]. The message emphasized that choosing PBMAs could contribute to reducing greenhouse gas emissions and land use and thereby contribute to climate-change mitigation and a more sustainable future. Because all respondents received identical information and no control or comparison condition was included, the environmental information was used to establish a standardized evaluation context rather than as an experimental manipulation. Accordingly, the independent effect of the environmental information cannot be estimated from the present design. The original Korean environmental information presented to respondents and its English translation are provided in Supplementary Section S3.

After reviewing the environmental information, respondents completed measures of gain, hedonic, and normative goals, followed by egoistic, hedonic, and biospheric values, attitude toward PBMAs, and demographic characteristics. This measurement sequence was informed by GFT and related value–goal research, which conceptualize values as relatively stable orientations and goal frames as more context-sensitive motivational states [12,27]. Accordingly, the goal-related measures were administered immediately after presentation of the common environmental information, whereas the relatively stable value orientations were measured subsequently. The present analyses focused on normative goals in accordance with the proposed research model. Because all focal measures were completed after exposure to the environmental information, the possibility that the information influenced the salience of environmental considerations and subsequent responses cannot be ruled out.

### 3.3. Measures

The focal study constructs were operationalized using items adapted from established scales or developed with reference to prior measures. All focal construct items were administered using a seven-point agreement scale ranging from 1 (“strongly disagree”) to 7 (“strongly agree”). Where the original measures employed different response formats, the response format was adapted to the seven-point scale used in the present study. Because the survey was administered in Korean, items originally developed in English were translated into Korean and reviewed by academic experts for clarity and conceptual equivalence. The Korean items were subsequently back-translated into English and compared with the original wording to assess semantic consistency.

Biospheric value and hedonic value were measured using four and three items, respectively, adapted from Yasir et al. [28], which operationalized the value dimensions proposed by Steg et al. [13]. In the present study, hedonic value refers to a general orientation toward enjoyment and personal gratification rather than PBMA-specific sensory pleasure or perceived taste.

Normative goals were conceptualized according to Goal-Framing Theory [11]. The normative goal measure initially comprised five PBMA-specific items (NG1–NG5), adapted or developed with reference to Onwezen [15], Puzzo and Prati [17], and the GFT conceptualization of normative goal. The items were designed to capture the perceived normative meaning of PBMA purchasing, including social recognition, responsibility, and appropriate conduct. This operationalization differed from the goal-importance format used by Onwezen [15] and should therefore be regarded as an adaptation rather than an equivalent reproduction of the original measure. One item (NG1) was excluded following a post hoc assessment of its conceptual content and measurement model diagnostics, resulting in a retained four-item scale (NG2–NG5). Although NG1 met conventional measurement criteria, its removal was informed by conceptual considerations and model diagnostics rather than failure to satisfy a predefined statistical retention threshold. The item-level sources and adaptation rationales are provided in Supplementary Section S2 (Table S2). The assessment of NG1 and comparison of the five- and four-item measurement models are presented in Table S3. CFA results for the five-item measurement model and sensitivity analyses of the main findings are reported in Tables S4 and S5, respectively.

Attitude toward PBMAs was measured using three items adapted from Park and Namkung [29]. Interdependent self-construal was assessed using the four-item short form validated by Hofmann et al. [30], which was derived from Singelis’s [19] interdependent self-construal scale. The items retained for the final analyses are presented in Supplementary Table S1.

### 3.4. Data Analysis

Data were analyzed using IBM SPSS Statistics 27 and AMOS 23. Descriptive statistics and frequency analyses were conducted to characterize the sample and examine variable distributions, and Pearson correlations were calculated to assess bivariate associations among the study constructs. Composite scores were calculated as the mean of the retained items for each construct. No missing values were identified in the variables used in the analyses; therefore, no missing-data imputation was required.

As described in Section 3.1, the final analytic dataset comprised 288 respondents following post hoc data screening. Because the exclusion procedure had not been prospectively specified, additional robustness analyses were conducted where feasible to evaluate the stability of the principal findings.

Confirmatory factor analysis (CFA) was conducted using AMOS 23 with maximum likelihood estimation to evaluate the measurement model. Model fit was assessed using χ^2^/df, the incremental fit index (IFI), the comparative fit index (CFI), the Tucker–Lewis index (TLI), and the root mean square error of approximation (RMSEA). Convergent validity was evaluated using standardized factor loadings and average variance extracted (AVE), construct reliability using composite reliability (CR), and discriminant validity using the heterotrait–monotrait ratio (HTMT) [31]. The five- and four-item normative goal models were compared as part of the evaluation of NG1, with detailed results reported in Supplementary Table S3. No parameters suggested by modification indices were added to the final measurement model.

Regression-based indirect-effect and moderation analyses were conducted using PROCESS macro version 4.1 for SPSS [32]. PROCESS Model 4 was used to examine the associations specified in H1, RQ1, and H2 and the indirect associations specified in H3 and H4. Biospheric and hedonic values were included in the relevant regression equations so that the association of each value was estimated while accounting for the other. Indirect associations were evaluated using 5000 bootstrap samples and 95% bootstrap confidence intervals.

To address RQ2, PROCESS Model 1 was used to examine whether interdependent self-construal moderated the association between hedonic value and normative goal, with biospheric value included as a covariate. Hedonic value and interdependent self-construal were mean-centered before calculation of the interaction term, and the significant interaction was probed using simple slopes at the mean and ±1 SD of interdependent self-construal. HC3 heteroscedasticity-consistent standard errors were used as a robustness check. Influence diagnostics based on Cook’s distance were additionally examined, and a sensitivity analysis excluding 13 potentially influential observations (N = 275) is reported in Supplementary Section S5.

Finally, PROCESS Model 7 was used to explore whether the indirect association between hedonic value and attitude toward PBMAs through normative goals varied across levels of interdependent self-construal. In this model, interdependent self-construal moderated the first-stage association between hedonic value and normative goal. Biospheric value was included as a covariate, and conditional indirect associations and the index of moderated mediation were estimated using 5000 bootstrap samples and 95% bootstrap confidence intervals. Because this analysis extended beyond the primary tests of H4 and RQ2, it was treated as exploratory.

As an additional sensitivity analysis, the indirect-effect, moderation, and conditional indirect-effect analyses were repeated after excluding the 41 respondents who selected “Never” on the separate PBMA consumption-frequency item from the final analytic sample (N = 247). The results are reported in Supplementary Section S4. To assess the robustness of the findings to the inclusion of NG1, PROCESS Models 4, 1, and 7 were re-estimated using the five-item normative goal measure (NG1–NG5). The results were compared with those obtained using the retained four-item measure (NG2–NG5), as reported in Supplementary Table S5.

## 4. Results

### 4.1. Descriptive Statistics and Measurement Model

Descriptive statistics and Pearson correlations among the study constructs are presented in Table 2. All constructs were significantly and positively correlated, with coefficients ranging from 0.259 to 0.718. Biospheric value (BV) was positively correlated with normative goal (NG) (r = 0.504, *p* < 0.001), and hedonic value (HV) was also positively correlated with NG (r = 0.353, *p* < 0.001). The strongest correlation was observed between NG and attitude toward PBMAs (ATT) (r = 0.718, *p* < 0.001).

The results of the confirmatory factor analysis (CFA) are presented in Table 3. The measurement model demonstrated acceptable fit: χ^2^ = 274.38, df = 125, χ^2^/df = 2.195, IFI = 0.961, TLI = 0.952, CFI = 0.961, and RMSEA = 0.065. Standardized factor loadings ranged from 0.610 to 0.938, and composite reliability (CR) values ranged from 0.796 to 0.918. The average variance extracted (AVE) values for HV, BV, NG, and ATT exceeded 0.50. The AVE for Inter-SC was 0.496, slightly below the conventional 0.50 threshold, whereas its CR was 0.796 and its standardized factor loadings ranged from 0.610 to 0.793. Thus, Inter-SC showed adequate construct reliability, although its convergent validity was somewhat weaker than that of the other constructs.

Discriminant validity was further assessed using the heterotrait–monotrait ratio of correlations (HTMT). As shown in Table 4, HTMT values ranged from 0.290 to 0.797 and were all below the conservative threshold of 0.85 [31]. The highest HTMT value was observed between normative goal and attitude toward PBMAs (0.797), but it remained below the 0.85 threshold. These results supported discriminant validity among the study constructs.

### 4.2. Direct and Indirect Associations

The direct and indirect associations among the study variables are presented in Table 5. BV was positively associated with NG (B = 0.542, *p* < 0.001), supporting H1. With respect to RQ1, HV was also positively associated with NG (B = 0.195, *p* < 0.05). Together, BV and HV explained 26.8% of the variance in NG (R^2^ = 0.268).

In the model with ATT as the outcome, NG was positively associated with ATT (B = 0.614, *p* < 0.001), supporting H2. After accounting for NG and the other value, BV remained positively associated with ATT (B = 0.270, *p* < 0.001), whereas the corresponding association between HV and ATT was not statistically significant (B = −0.107, *p* > 0.05). The model explained 54.8% of the variance in ATT (R^2^ = 0.548).

Bootstrap analyses based on 5000 resamples showed a significant indirect association between BV and ATT through NG (indirect effect = 0.333, bootstrap SE = 0.056, 95% CI [0.226, 0.451]), supporting H3. The indirect association between HV and ATT through NG was also significant (indirect effect = 0.120, bootstrap SE = 0.045, 95% CI [0.029, 0.210]), supporting H4. To further contextualize the direct and indirect associations, the corresponding total association estimates with ATT were B = 0.603 for BV and B = 0.013 for HV. Because the data were cross-sectional, these indirect associations should not be interpreted as evidence of causal mediation.

### 4.3. Moderating Role of Interdependent Self-Construal

To address RQ2, the moderating role of interdependent self-construal (Inter-SC) in the association between hedonic value (HV) and normative goal (NG) was examined with biospheric value (BV) included as a covariate. The HV × Inter-SC interaction was statistically significant (B = 0.262, SE = 0.086, *p* = 0.003, 95% CI [0.093, 0.431]) and accounted for an additional 2.3% of the variance in NG (ΔR^2^ = 0.023, ΔF = 9.290, *p* = 0.003). Thus, the association between HV and NG varied across levels of Inter-SC.

Simple slope analyses showed that the association between HV and NG was not statistically significant at a low level of Inter-SC (−1 SD; B = 0.051, SE = 0.097, *p* = 0.599, 95% CI [−0.140, 0.242]). The association was positive and statistically significant at the mean level of Inter-SC (B = 0.261, SE = 0.095, *p* = 0.006, 95% CI [0.075, 0.448]) and at a high level of Inter-SC (+1 SD; B = 0.472, SE = 0.135, *p* = 0.001, 95% CI [0.207, 0.737]). These findings indicate that, in response to RQ2, the HV–NG association was conditional on the level of Inter-SC. The moderation and simple slope results are presented in Table 6. Robustness analyses addressing the relatively high HV scores and potentially influential observations are reported in Supplementary Section S5.

### 4.4. Exploratory Conditional Indirect Associations

To further examine H4 and RQ2 jointly, the conditional indirect association between hedonic value (HV) and attitude toward PBMAs (ATT) through normative goal (NG) was explored across levels of interdependent self-construal (Inter-SC), with biospheric value (BV) included as a covariate. At a low level of Inter-SC (−1 SD), the conditional indirect association was not statistically significant (indirect effect = 0.031, bootstrap SE = 0.053, 95% CI [−0.077, 0.131]). At the mean level of Inter-SC, the indirect association was statistically significant (indirect effect = 0.161, bootstrap SE = 0.055, 95% CI [0.058, 0.275]), as it was at a high level of Inter-SC (+1 SD) (indirect effect = 0.290, bootstrap SE = 0.084, 95% CI [0.142, 0.473]).

The index of moderated mediation was 0.161 (bootstrap SE = 0.054, 95% CI [0.070, 0.284]), with the confidence interval excluding zero. These results indicate that, after accounting for BV, the magnitude of the indirect association between HV and ATT through NG varied across levels of Inter-SC. Because this conditional indirect analysis extended beyond the primary tests of H4 and RQ2, it was treated as exploratory and should not be interpreted as evidence of a causal moderated mediation process. The results of the four-item exploratory conditional indirect-effect analysis are presented in Table 7.

Sensitivity analyses using the five-item normative goal measure (NG1–NG5) yielded results consistent with those obtained using the retained four-item measure (NG2–NG5). The indirect associations of biospheric and hedonic values with attitude through normative goal remained statistically significant, as did the interaction between hedonic value and interdependent self-construal. The index of moderated mediation also remained statistically significant, and the pattern of conditional indirect associations was unchanged. These findings suggest that the main results were robust to the inclusion of NG1 (Supplementary Table S5).

## 5. Discussion

### 5.1. Theoretical Implications

This study extends the application of Goal-Framing Theory (GFT) to the PBMA context by examining associations among relatively stable value orientations, normative goal, and attitude toward PBMAs, as well as the moderating role of interdependent self-construal.

First, biospheric value was positively associated with normative goal, consistent with the theoretical affinity between environmentally oriented values and a normative orientation toward appropriate action [11,12]. Previous research has linked biospheric value to environmental self-identity and personal norms [8,10]; however, these constructs are conceptually distinct from the GFT-based normative goal. The present findings therefore extend this literature by examining the association between biospheric value and normative goal specifically within the PBMA context.

Second, with respect to RQ1, hedonic value was positively associated with normative goals. This finding is notable given previous evidence that hedonic value may be negatively associated with environmentally relevant attitudes and behaviors [13]. Importantly, hedonic value in the present study reflects a general orientation toward enjoyment and personal gratification rather than PBMA-specific sensory pleasure or perceived taste. The finding therefore suggests that a general hedonic value orientation is not necessarily incompatible with normative goals in the environmental-information context examined here.

Third, normative goal was positively associated with attitude toward PBMAs, and both biospheric and hedonic values showed significant indirect associations with attitude through normative goal. These findings are consistent with the GFT perspective that relatively stable value orientations may be associated with more context-sensitive motivational goals, which are in turn associated with situation-specific evaluations [11,14]. However, given the cross-sectional design, these indirect associations should not be interpreted as evidence of causal mediation.

Fourth, the association between hedonic value and normative goal varied across levels of interdependent self-construal. The association was not statistically significant at a low level of interdependent self-construal but was positive at the mean and high levels. Although social norms and the GFT-based normative goal are conceptually distinct, previous research suggests that self-construal can condition norm-related responses in pro-environmental contexts [20,21]. The present findings therefore suggest that interdependent self-construal may represent a boundary condition in value–goal associations. The conditional indirect analysis showed a similar pattern but should be regarded as exploratory.

### 5.2. Practical Implications

The findings suggest that consumers with a stronger general orientation toward enjoyment and personal gratification should not necessarily be assumed to be less aligned with normative environmental considerations related to PBMAs. Within the common environmental-information context examined in this study, hedonic value was positively associated with normative goal. Sustainability-oriented PBMA communication may therefore benefit from recognizing that pleasure-oriented value orientations and normative environmental considerations are not necessarily incompatible.

However, the present study did not measure PBMA-specific sensory pleasure or experimentally compare alternative communication strategies. The findings therefore do not demonstrate that combining hedonic or sensory appeals with environmental messages would improve consumer responses. Future experimental research should directly compare such communication strategies before specific marketing recommendations are made.

The moderating role of interdependent self-construal further suggests that relational self-orientation may be relevant to understanding heterogeneity in consumer responses. Nevertheless, because segmentation and targeting strategies were not experimentally tested, these findings should be regarded as a basis for future research rather than as evidence of the effectiveness of a specific targeting approach.

### 5.3. Limitations and Future Research

This study has several limitations. First, the cross-sectional design does not permit causal inference among the study variables. In addition, all respondents received the same environmental information before completing the focal measures, and no control or comparison condition was included. Therefore, the independent influence of the environmental information on subsequent responses cannot be determined. Future research should employ longitudinal or experimental designs with appropriate comparison conditions to clarify causal and contextual mechanisms.

Second, the study focused on Korean consumers aged 20–39 recruited through an online panel and relied on self-reported measures. Generalization to other age groups, cultural contexts, and actual purchasing behavior should therefore be made cautiously. Because the focal constructs were measured within the same survey, common method variance also cannot be entirely ruled out. In addition, the data-screening procedure leading to the final analytic sample of 288 respondents was conducted post hoc rather than on the basis of prospectively specified data-quality exclusion criteria. This should be considered when interpreting the findings, and future studies should establish and report data-quality screening criteria prospectively.

Third, eligibility was based on prior consumption experience, whereas PBMA consumption frequency was assessed separately. Although sensitivity analyses excluding respondents who selected “Never” yielded substantively consistent findings, the difference between the screening criterion and the subsequent frequency measure may have introduced ambiguity. Future studies should use screening and consumption-frequency measures with clearly aligned reference periods.

Fourth, the normative goal measure was adapted from prior measures and differed from the original goal-importance format used by Onwezen [15]. NG1 was excluded following a post hoc assessment of its conceptual content and measurement model diagnostics, and the retained item concerning environmental commitment and setting an example may overlap conceptually with moral goal framing. Although sensitivity analyses indicated that the principal findings were robust to the inclusion of NG1, the post hoc nature of the item-removal decision warrants further validation of the normative goal measure in independent samples, particularly with respect to its discriminant validity alongside related constructs such as moral goals, personal norms, social norms, and environmental self-identity. In addition, the AVE for interdependent self-construal was slightly below the conventional 0.50 threshold, suggesting somewhat weaker convergent validity than for the other constructs.

Finally, hedonic value scores were concentrated toward the upper end of the scale, suggesting a potential ceiling effect. Although robustness analyses using HC3 standard errors and influence diagnostics supported the stability of the moderation finding, replication using alternative measures with greater sensitivity at the upper end of the scale and more diverse samples is warranted. The exploratory conditional indirect analysis should likewise be replicated before stronger conclusions are drawn.

## 6. Conclusions

This study examined how biospheric and hedonic values are associated with normative goals and attitudes toward PBMAs and whether interdependent self-construal conditions the hedonic value–normative goal association. Both biospheric and hedonic values were positively associated with normative goals, which were positively associated with attitude toward PBMAs, and both values showed significant indirect associations with attitude through normative goals. The hedonic value–normative goal association also varied across levels of interdependent self-construal. These findings suggest that a general hedonic value orientation is not necessarily incompatible with normative goals in the environmental-information context examined in this study and that interdependent self-construal may represent a relevant boundary condition in value–goal associations. From a practical perspective, the findings suggest that sustainability-oriented PBMA communication should not assume that consumers with stronger general hedonic value orientations are necessarily less receptive to normative environmental considerations; however, this implication requires experimental validation. Further experimental and longitudinal research is needed to clarify the causal and contextual mechanisms underlying these findings.

## Figures and Tables

**Table 1:** Sample characteristics.

Variable	Category	N	%
Gender	Male	145	50.3
	Female	143	49.7
Age	20–29 years	131	45.5
	30–39 years	157	54.5
Education	High school or below	21	7.3
	Two-year college	36	12.5
	Four-year university	197	68.4
	Graduate school or above	34	11.8
PBMA consumption frequency	Daily	6	2.1
	2–3 times per week or more	42	14.6
	Once a week	50	17.4
	Once every two weeks	22	7.6
	Once a month	28	9.7
	Once every six months	20	6.9
	Once a year or less	6	2.1
	Rarely	73	25.3
	Never	41	14.2
Total		288	100.0

Note. PBMA = plant-based milk alternative. PBMA consumption frequency was assessed separately from the eligibility screening question. Eligibility was based on prior consumption of plant-based beverages identified during screening. Respondents who selected “Never” on the separate consumption-frequency item were retained in the primary analysis because they had satisfied the prior-consumption screening criterion. Sensitivity analyses excluding these respondents (N = 247) are reported in Supplementary Section S4.

**Table 2:** Descriptive statistics and correlations among study constructs.

Construct	Mean	SD	HV	BV	NG	ATT	Inter-SC
HV	6.030	0.833	1.000				
BV	5.465	0.965	0.499 ***	1.000			
NG	4.304	1.199	0.353 ***	0.504 ***	1.000		
ATT	4.565	1.165	0.259 ***	0.505 ***	0.718 ***	1.000	
Inter-SC	5.287	0.803	0.513 ***	0.446 ***	0.301 ***	0.339 ***	1.000

Note. N = 288. HV = hedonic value; BV = biospheric value; NG = normative goal; ATT = attitude toward PBMAs; Inter-SC = interdependent self-construal. *** *p* < 0.001.

**Table 3:** Confirmatory factor analysis and construct reliability.

Construct/Item	β	B	SE	SMC	AVE	CR
HV1	0.842	1	—	0.708	0.770	0.909
HV2	0.879	1.002	0.054	0.772		
HV3	0.910	1.110	0.057	0.828		
BV1	0.879	1	—	0.772	0.740	0.918
BV2	0.680	0.876	0.065	0.462		
BV3	0.926	1.026	0.044	0.857		
BV4	0.932	0.998	0.042	0.869		
NG2	0.792	1	—	0.627	0.705	0.905
NG3	0.848	1.073	0.067	0.719		
NG4	0.818	1.040	0.068	0.670		
NG5	0.898	1.186	0.069	0.807		
ATT1	0.938	1	—	0.881	0.763	0.906
ATT2	0.882	1.070	0.047	0.779		
ATT3	0.795	0.939	0.051	0.633		
Inter-SC1	0.610	1	—	0.372	0.496	0.796
Inter-SC2	0.793	1.311	0.135	0.628		
Inter-SC3	0.755	1.302	0.137	0.569		
Inter-SC4	0.644	1.053	0.123	0.415		

Note. HV = hedonic value; BV = biospheric value; NG = normative goal; ATT = attitude toward PBMAs; Inter-SC = interdependent self-construal; β = standardized factor loading; B = unstandardized factor loading; SE = standard error; SMC = squared multiple correlation; AVE = average variance extracted; CR = composite reliability. The final normative goal construct comprised NG2–NG5; NG1 was excluded following the measurement evaluation. The five- and four-item model comparison is reported in Supplementary Table S3. Model fit: χ^2^ = 274.38, df = 125, χ^2^/df = 2.195, *p* < 0.001, IFI = 0.961, TLI = 0.952, CFI = 0.961, RMSEA = 0.065.

**Table 4:** Discriminant validity assessed using HTMT.

Construct	HV	BV	NG	ATT	Inter-SC
HV	—				
BV	0.550	—			
NG	0.390	0.560	—		
ATT	0.290	0.550	0.797	—	
Inter-SC	0.600	0.530	0.360	0.400	—

Note. HTMT = heterotrait-monotrait ratio of correlations. All HTMT values were below the conservative threshold of 0.85, supporting discriminant validity among the constructs.

**Table 5:** Results of Direct and Indirect Association Analyses

	Path	B	SE	Boot SE	t	95% CI	F	R^2^
Path coefficients	BV → NG	0.542	0.073		7.463 ***	—	52.142	0.268
	HV → NG	0.195	0.084		2.317 *	—		
	BV → ATT	0.270	0.061		4.450 ***	—	114.607	0.548
	HV → ATT	−0.107	0.065		−1.642	—		
	NG → ATT	0.614	0.045		13.558 ***	—		
Indirect associations	BV → NG → ATT	0.333		0.056	—	[0.226, 0.451]	—	—
	HV → NG → ATT	0.120		0.045	—	[0.029, 0.210]		

Note. BV = biospheric value; HV = hedonic value; NG = normative goal; ATT = attitude toward PBMAs. B denotes the unstandardized regression coefficient for direct associations and the unstandardized indirect-effect estimate for indirect associations. SE denotes the standard error of regression coefficients; Boot SE denotes the bootstrap standard error of indirect effects. Indirect associations were estimated using 5000 bootstrap samples. Confidence intervals excluding zero were considered statistically significant. * *p* < 0.05, *** *p* < 0.001.

**Table 6:** Moderation analysis and simple slopes.

Panel/Predictor	B	SE	t	*p*	95% CI	R^2^	ΔR^2^	ΔF
**Panel A. Moderation model controlling for BV**						0.293	0.023	9.290
HV	0.261	0.095	2.753	0.006	[0.075, 0.448]			
Inter-SC	0.055	0.090	0.607	0.545	[−0.123, 0.233]			
HV × Inter-SC	0.262	0.086	3.048	0.003	[0.093, 0.431]			
BV (control)	0.538	0.074	7.246	<0.001	[0.392, 0.684]			
**Panel B. Simple slopes of HV at levels of Inter-SC**								
Low (−1 SD)	0.051	0.097	0.527	0.599	[−0.140, 0.242]			
Mean	0.261	0.095	2.753	0.006	[0.075, 0.448]			
High (+1 SD)	0.472	0.135	3.502	0.001	[0.207, 0.737]			

Note. HV = hedonic value; BV = biospheric value; Inter-SC = interdependent self-construal; NG = normative goal. Unstandardized coefficients (B) are reported. HV and Inter-SC were mean-centered before computing the interaction term. BV was included as a covariate. ΔR^2^ represents the increase in explained variance attributable to the HV × Inter-SC interaction; ΔF represents the corresponding F-change. Simple slopes were estimated at the mean and ±1 SD of Inter-SC. NG was the dependent variable in the moderation analysis.

**Table 7:** Conditional indirect associations and index of moderated mediation controlling for biospheric value.

Path/Statistic	Inter-SC	Effect	Boot SE	95% CI
**HV** **→ NG → ATT**				
	Low (−1 SD)	0.031	0.053	[−0.077, 0.131]
	Mean	0.161	0.055	[0.058, 0.275]
	High (+1 SD)	0.290	0.084	[0.142, 0.473]
Index of moderated mediation	Inter-SC	0.161	0.054	[0.070, 0.284]

Note. HV = hedonic value; NG = normative goal; ATT = attitude toward PBMAs; Inter-SC = interdependent self-construal; BV = biospheric value. Conditional indirect associations were estimated at the mean and ±1 SD of Inter-SC, with BV included as a covariate. Boot SE denotes the bootstrap standard error. Bootstrap confidence intervals were based on 5000 bootstrap samples; intervals excluding zero were considered statistically significant.

## Data Availability

The data supporting the findings of this study are available from the corresponding author upon reasonable request.

## Supplementary Materials

The following supporting information can be downloaded at https://www.mdpi.com/article/10.3390/foods15213864/s1, Table S1: Measurement items; Table S2: Comparison of original and adapted normative goal items; Table S3: Comparison of five- and four-item normative goal measurement models; Figure S1: Environmental information presented to respondents; Table S4: Confirmatory factor analysis results for the five-item normative goal model; Table S5: Sensitivity analysis of the main findings with and without NG1; Table S6: Comparison of main analysis results before and after excluding respondents who selected “Never” for PBMA consumption frequency; Table S7: Robustness checks for the moderation analysis of interdependent self-construal in the association between hedonic value and normative goal; Figure S2: Interaction between hedonic value and interdependent self-construal in predicting normative goals.

## References

[1] Poore J., Nemecek T. (2018). Reducing food’s environmental impacts through producers and consumers. Science.

[2] Jaeger S.R., Giacalone D. (2021). Barriers to consumption of plant-based beverages: A comparison of product users and non-users on emotional, conceptual, situational, conative and psychographic variables. Food Res. Int..

[3] Giacalone D., Clausen M.P., Jaeger S.R. (2022). Understanding barriers to consumption of plant-based foods and beverages: Insights from sensory and consumer science. Curr. Opin. Food Sci..

[4] Halme M., Pirttilä-Backman A.-M., Pham T. (2023). The perceived value of oat milk and the food-choice motives of young, urban people. Br. Food J..

[5] Alsado C., Chen L., Wismer W. (2025). Are plant-based milks unique products or dairy milk substitutes? A study of consumer perceptions, uses, and consumption motivations of plant-based and dairy milks. Food Qual. Prefer..

[6] Onwezen M.C., Bouwman E.P., Reinders M.J., Dagevos H. (2021). A systematic review on consumer acceptance of alternative proteins: Pulses, algae, insects, plant-based meat alternatives, and cultured meat. Appetite.

[7] Matharu G.K., von der Heidt T., Sorwar G. (2024). Consumer behavior toward plant-based foods: A theoretical review, synthesis and conceptual framework. Br. Food J..

[8] van der Werff E., Steg L., Keizer K. (2013). The value of environmental self-identity: The relationship between biospheric values, environmental self-identity and environmental preferences, intentions and behaviour. J. Environ. Psychol..

[9] Isbanner S., Fechner D., Attwood S. (2025). Goal-framing theory and sustainable food choices: Leveraging spillover to activate moral goals. Appetite.

[10] Wang S.-T., Chang S.-C. (2026). Influence of altruistic and biospheric values on vegetarians’ intentions to repurchase plant-based foods. Br. Food J..

[11] Lindenberg S., Steg L. (2007). Normative, gain and hedonic goal frames guiding environmental behavior. J. Soc. Issues.

[12] Steg L., Bolderdijk J.W., Keizer K., Perlaviciute G. (2014). An integrated framework for encouraging pro-environmental behaviour: The role of values, situational factors and goals. J. Environ. Psychol..

[13] Steg L., Perlaviciute G., van der Werff E., Lurvink J. (2014). The significance of hedonic values for environmentally relevant attitudes, preferences, and actions. Environ. Behav..

[14] Steg L., Lindenberg S., Keizer K. (2016). Intrinsic motivation, norms and environmental behaviour: The dynamics of overarching goals. Int. Rev. Environ. Resour. Econ..

[15] Onwezen M.C. (2023). Goal-framing theory for sustainable food behaviour: The added value of a moral goal frame across different contexts. Food Qual. Prefer..

[16] Erhard A., Jahn S., Boztug Y. (2024). Tasty or sustainable? Goal conflict in plant-based food choice. Food Qual. Prefer..

[17] Puzzo G., Prati G. (2025). Unraveling the influence of convenience situational factors on e-waste recycling behaviors: A goal-framing theory approach. Sustain. Dev..

[18] Markus H.R., Kitayama S. (1991). Culture and the self: Implications for cognition, emotion, and motivation. Psychol. Rev..

[19] Singelis T.M. (1994). The measurement of independent and interdependent self-construals. Pers. Soc. Psychol. Bull..

[20] Saracevic S., Schlegelmilch B.B. (2021). The impact of social norms on pro-environmental behavior: A systematic literature review of the role of culture and self-construal. Sustainability.

[21] Hu X., Hu J. (2025). Exploring the mechanisms of different social norms influencing green consumption attitude and behavior. Asia Pac. J. Mark. Logist..

[22] Stern P.C., Dietz T. (1994). The value basis of environmental concern. J. Soc. Issues.

[23] de Groot J.I.M., Steg L. (2008). Value orientations to explain beliefs related to environmental significant behavior: How to measure egoistic, altruistic, and biospheric value orientations. Environ. Behav..

[24] Khan K., Hameed I., Akram U., Hussainy S.K. (2023). Do normative triggers and motivations influence the intention to purchase organic food? An application of the goal-framing theory. Br. Food J..

[25] Ha A.W., Lee M.H., Kim S.H. (2023). Consumption propensity of Korean adults and nutrient contents of domestic commercial products for plant-based milk alternatives. J. Korean Soc. Food Sci. Nutr..

[26] Khanpit V., Viswanathan S., Hinrichsen O. (2024). Environmental impact of animal milk vs plant-based milk: Critical review. J. Clean. Prod..

[27] Lindenberg S., Steg L., van Trijp H.C.M. (2013). Goal-framing theory and norm-guided environmental behavior. Encouraging Sustainable Behavior: Psychology and the Environment.

[28] Yasir N., Mahmood N., Mehmood H.S., Rashid O., Liren A. (2021). The integrated role of personal values and theory of planned behavior to form a sustainable entrepreneurial intention. Sustainability.

[29] Park C.-I., Namkung Y. (2024). Consumer values, attitudes, and behavior towards plant-based alternatives. Foods.

[30] Hofmann V., Schwayer L.M., Stokburger-Sauer N.E., Wanisch A.T. (2021). Consumers’ self-construal: Measurement and relevance for social media communication success. J. Consum. Behav..

[31] Henseler J., Ringle C.M., Sarstedt M. (2015). A new criterion for assessing discriminant validity in variance-based structural equation modeling. J. Acad. Mark. Sci..

[32] Hayes A.F. (2022). Introduction to Mediation, Moderation, and Conditional Process Analysis: A Regression-Based Approach.

