# Supplementary material for: Hedonic Value and Normative Goal: The Role of Interdependent Self-Construal in the Evaluation of Plant-Based Milk Alternatives
Source: Foods. 2026 Oct 28;15(21):3864. doi: 10.3390/foods15213864 (PMC13762790; doi:10.3390/foods15213864)
Supplement: Supplementary file 1 [file foods-15-03864-s001.zip › foods-4643387-supplementary.pdf]

Supplementary Material

Index

S1. Measurement

S2. Adaptation of Normative Goal Items

S3. Environmental Information Presented to Respondents

S4. Sensitivity Analysis

S5. Ceiling Effect Assessment and Robustness Checks of the Moderation Analysis

S1. Measurement

Table S1. Measurement items.

Subscale	Item code	Items	References
Hedonic value	HV1	It is important to experience joy in life.	[13,28]
	HV2	It is important to enjoy life.	
	HV3	It is important to feel personal gratification.	
Biospheric value	BV1	It is important to respect the Earth.	
	BV2	It is important to feel connected with nature.	
	BV3	It is important to protect the environment.	
	BV4	It is important to prevent pollution.	
Normative goal	NG2	Purchasing plant-based milk alternatives is recognized by those who value the environment.	[11,15,17]
	NG3	I would feel a sense of responsibility if I purchased plant-based milk alternatives.	
	NG4	Purchasing plant-based milk alternatives shows others that I am making the right choice.	
	NG5	Purchasing plant-based milk alternatives demonstrates commitment to protecting the environment and setting an example.	
Attitude	ATT1	Choosing PBMAs is a wise decision.	[29]
	ATT2	Choosing PBMAs is satisfying.	
	ATT3	PBMAs are trustworthy.	
Interdependent Self-Construal	Inter-SC1	I respect authority figures with whom I interact.	[19,30]
	Inter-SC2	Maintaining harmony within my group is important to me.	
	Inter-SC3	I respect people who are modest about themselves.	
	Inter-SC4	It is important to respect decisions made by the group.	

Note. PBMAs = plant-based milk alternatives. Item codes correspond to those used in the measurement analysis. NG1 was excluded from the final normative goal scale and is presented in Table S2 for transparency. The adaptation of the normative goal items and their conceptual correspondence with Onwezen [15] are described in Section S2.

S2. Adaptation of Normative Goal Items

In goal-framing theory, normative goals direct attention to what is appropriate or ought to be done in a given situation [11]. This orientation is not limited to seeking social approval but also concerns responsibility and appropriate conduct. Onwezen [15] assessed normative goal frames in food choice through reference-group values, social recognition, and the demonstration of appropriate behavior, while distinguishing normative from moral goal frames.

Empirical research directly examining the relationships between values and goal frames remains limited, particularly in food-choice settings. The present study therefore drew on both Onwezen [15] and Puzzo and Prati [17]. Onwezen provided food-choice-specific items reflecting social expectations and appropriate behavior, whereas Puzzo and Prati examined the relationship between values and goal frames in electronic waste recycling and included perceived responsibility as an indicator of normative goals. Although the behavioral contexts differ, both studies informed the adaptation of items to PBMA purchasing, where social expectations and considerations of responsible conduct may be relevant.

Unlike Onwezen [15], who assessed the importance of goals in food choice, the present study measured the perceived normative meaning of purchasing PBMAs. This change in measurement focus means that the adapted items should not be regarded as equivalent to the original instruments. Furthermore, while Lindenberg and Steg [11] described normative goals as one of three overarching goal frames, Onwezen [15] distinguished normative and moral goal frames in food choice. The item concerning environmental commitment and setting an example may reflect aspects of both. The adapted measure was therefore interpreted within the specific context of PBMA purchasing, with attention to this conceptual overlap. The sources and modifications of individual items are presented in Table S2.

Table S2. Comparison of original and adapted normative goal items.

Item code	Original item or conceptual source	Adapted item	Rationale for adaptation
NG1[a]	Onwezen [15], Item 17: Alignment with family and friends' values	Purchasing plant-based milk alternatives aligns with the values of my family and friends.	Applied reference-group values to PBMA purchasing to assess perceived alignment with significant others' expectations.
NG2	Onwezen [15], Item 18: Approval by environmentally concerned others	Purchasing plant-based milk alternatives is recognized by those who value the environment.	Adapted social recognition to PBMA purchasing, reflecting the perceived approval of environmentally responsible behavior.
NG3	Puzzo and Prati [17]: Responsibility associated with electronic waste recycling	I would feel a sense of responsibility if I purchased plant-based milk alternatives.	Adapted the responsibility-related item from recycling to PBMA purchasing while retaining its focus on responsible conduct within the normative goal frame.
NG4	Onwezen [15], Item 19: Demonstrating	Purchasing plant-based milk alternatives shows	Applied the expression of appropriate behavior to

	appropriate behavior to others	others that I am making the right choice.	PBMA purchasing, emphasizing socially recognized standards of conduct.
NG5[b]	Lindenberg and Steg [11]: Normative goal framing; Onwezen [15]: Normative and moral goal concepts	Purchasing plant-based milk alternatives demonstrates commitment to protecting the environment and setting an example.	Linked environmental commitment with setting an example for others, reflecting both moral concern and the social expression of appropriate behavior.

Note. PBMAs = plant-based milk alternatives; NG = normative goal. Original item concepts are summarized from the cited studies. [a]NG1 was excluded from the final measure; supporting analyses are reported below. [b]The references for NG5 indicate its conceptual basis rather than a directly corresponding original item.

NG1 was further evaluated in terms of its conceptual content and measurement properties (Table S3). NG1 assessed the perceived alignment of PBMA purchasing with the values of family and friends, whereas NG2–NG5 primarily reflected social recognition, perceived responsibility, and environmentally appropriate conduct. Although NG1 captured a social aspect of normative goals, its emphasis on reference-group value alignment differed conceptually from the remaining items.

NG1 exhibited an acceptable standardized factor loading (.771), and both the five- and four-item measurement models satisfied the specified HTMT criterion of .85. The four-item model showed more favorable descriptive fit indices than the five-item model (CFI = .961 vs. .947; TLI = .952 vs. .936; RMSEA = .065 vs. .073). The latent correlation between normative goal and attitude decreased from .830 to .794, while HTMT decreased from .841 to .797. Accordingly, the removal of NG1 was a post hoc decision informed by item content and measurement model diagnostics, rather than a failure to meet a predefined statistical retention threshold.

To assess the implications of this decision, the five-item measurement model was further evaluated using confirmatory factor analysis (Table S4), and the primary analyses were repeated with all five normative-goal items included (Table S5). The sensitivity analyses indicated that the direction and statistical significance of the principal findings remained consistent across the four- and five-item specifications, supporting the robustness of the findings to the inclusion of NG1.

Table S3. Comparison of five- and four-item normative goal measurement models.

Measure	NG1 included	NG1 excluded
Model fit		
χ^2	359.313	274.380
df	142	125
χ^2/df	2.530	2.195
CFI	.947	.961
TLI	.936	.952
IFI	.947	.961
RMSEA	.073	.065
Measurement properties		
NG1 standardized factor loading	.771	—
NG–ATT latent correlation	.830	.794
NG–ATT HTMT	.841	.797

Note. CFA = confirmatory factor analysis; NG = normative goal; ATT = attitude toward PBMAs; CFI = comparative fit index; TLI = Tucker–Lewis index; IFI = incremental fit index; RMSEA = root mean square error of approximation; HTMT = heterotrait–monotrait ratio. Both measurement models included hedonic value, biospheric value, normative goal, attitude, and interdependent self-construal (N = 288). The models differed in the number of normative-goal indicators and were compared descriptively. Both specifications satisfied the specified HTMT criterion of .85.

S3. Environmental Information Presented to Respondents

All respondents were presented with the same environmental information before completing the survey measures. Figure S1 shows the original Korean material used in the survey. An English translation of the environmental message is provided below.

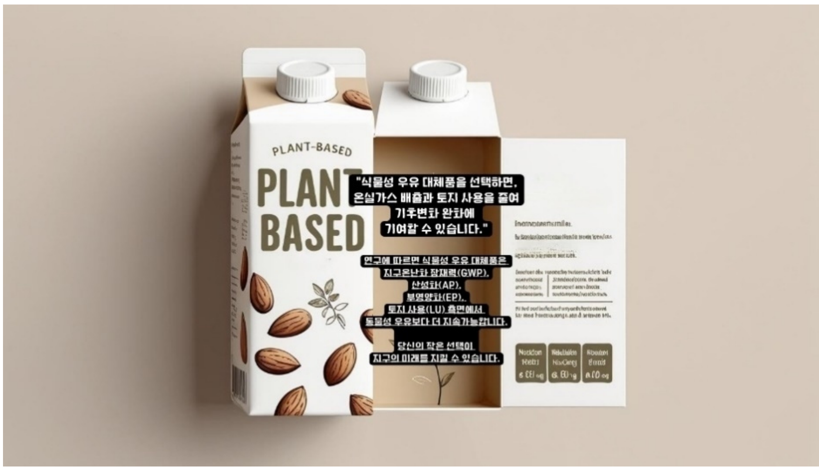

Figure S1. Environmental information presented to respondents (original Korean version).

English Translation of the Environmental Information

By choosing plant-based milk, you can help reduce greenhouse gas emissions and land use, contributing to climate change mitigation. Research shows that plant-based milk is more sustainable than dairy milk in terms of global warming potential (GWP), acidification (AP), eutrophication (EP), and land use (LU). Your small choice can help protect the future of our planet.

S4. Sensitivity Analysis

S4.1. Sensitivity Analysis for the Inclusion of NG1

To assess the robustness of the findings to the inclusion of NG1, the five-item normative-goal measure (NG1–NG5) was evaluated using confirmatory factor analysis (Table S4). The measurement model included hedonic value, biospheric value, normative goal, attitude toward PBMAs, and interdependent self-construal. The five-item model demonstrated acceptable fit (χ^2 = 359.313, df = 142, CFI = .947, TLI = .936, IFI = .947, RMSEA = .073). NG1 exhibited an acceptable standardized factor loading (.771), while the five-item normative-goal construct showed satisfactory composite reliability (CR = .912) and average variance extracted (AVE = .675). Together with the HTMT results reported in Table S3, these findings indicate that NG1 satisfied conventional measurement criteria and that the five-item specification was an acceptable alternative measurement model.

The primary analyses were subsequently repeated using the five-item normative-goal measure and compared with those based on the retained four-item measure (NG2–NG5) (Table S5). In PROCESS Model 4, the indirect association between biospheric value and attitude through normative goal remained statistically significant in both the four-item (estimate = .333, 95% bootstrap CI [.226, .451]) and five-item specifications (estimate = .363, 95% bootstrap CI [.254, .485]). Likewise, the indirect association between hedonic value and attitude through normative goal remained statistically significant in the four-item (estimate = .120, 95% bootstrap CI [.029, .210]) and five-item specifications (estimate = .132, 95% bootstrap CI [.034, .229]).

The moderation and exploratory conditional indirect-effect results were also consistent across the two specifications. In PROCESS Model 1, the interaction between hedonic value and interdependent self-construal was statistically significant using both the four-item (B = .262, p = .003, ΔR^2 = .023) and five-item measures (B = .261, p = .002, ΔR^2 = .025). In PROCESS Model 7, the conditional indirect association between hedonic value and attitude through normative goal was not statistically significant at low levels of interdependent self-construal but was statistically significant at the mean and high levels in both specifications. The index of moderated mediation was .161 (95% bootstrap CI [.070, .284]) for the four-item measure and .181 (95% bootstrap CI [.077, .311]) for the five-item measure, with both confidence intervals excluding zero.

Overall, retaining NG1 did not alter the direction or statistical significance of the principal indirect associations, the interaction effect, or the exploratory conditional indirect associations. These results support the robustness of the principal findings to the inclusion of NG1, although they do not establish the superiority of either measurement specification.

Table S4. Confirmatory factor analysis results for the five-item normative goal model.

	β	B	SE	SMC	AVE	CR
Hedonic value						
HV1	.842	1.000	—	.709		
HV2	.879	1.003	.054	.773	.769	.909
HV3	.909	1.109	.057	.827		

Biospheric value						
BV1	.879	1.000	—	.773	.740	.918
BV2	.680	.876	.064	.463		
BV3	.925	1.025	.043	.857		
BV4	.932	.998	.042	.868		
Normative goal (5 items)						
NG1	.771	1.000	—	.594	.675	.912
NG2	.784	1.065	.075	.615		
NG3	.853	1.159	.074	.727		
NG4	.818	1.117	.075	.668		
NG5	.877	1.244	.077	.769		
Attitude						
ATT1	.938	1.000	—	.880	.763	.906
ATT2	.883	1.071	.046	.780		
ATT3	.794	.938	.050	.631		
Interdependent self-construal						
Inter-SC1	.610	1.000	—	.372	.496	.796
Inter-SC2	.793	1.311	.135	.629		
Inter-SC3	.755	1.303	.137	.570		
Inter-SC4	.644	1.052	.123	.414		

Note. N = 288. β = standardized factor loading; B = unstandardized factor loading; SE = standard error; SMC = squared multiple correlation; AVE = average variance extracted; CR = composite reliability. HV = hedonic value; BV = biospheric value; NG = normative goal; ATT = attitude toward PBMAs; Inter-SC = interdependent self-construal. Model fit: $\chi^2 = 359.313$, df = 142, $\chi^2/df = 2.530$, $p < .001$, IFI = .947, TLI = .936, CFI = .947, RMSEA = .073.

Table S5. Sensitivity analysis of the main findings with and without NG1.

Path / Statistic	NG1 excluded (4-item NG)	NG1 included (5-item NG)
	Indirect associations (Model 4)	
BV → NG → ATT	.333 [.226, .451]	.363 [.254, .485]
HV → NG → ATT	.120 [.029, .210]	.132 [.034, .229]
	Moderation analysis (Model 1)	
HV × Inter-SC (B)	.262	.261
p	.003	.002
ΔR^2	.023	.025
	Exploratory conditional indirect associations (Model 7)	
	HV → NG → ATT	
Low Inter-SC (−1 SD)	.031 [−.077, .131]	.031 [−.091, .142]
Mean Inter-SC	.161 [.058, .275]	.176 [.066, .295]
High Inter-SC (+1 SD)	.290 [.142, .473]	.322 [.161, .513]
Index of moderated mediation	.161 [.070, .284]	.181 [.077, .311]

Note. BV = biospheric value; HV = hedonic value; NG = normative goal; ATT = attitude toward PBMAs; Inter-SC = interdependent self-construal; B = unstandardized regression coefficient; CI = confidence interval. The four-item normative-goal measure comprises NG2–NG5, whereas the five-item measure comprises NG1–NG5. All analyses used the same sample (N = 288) and identical model specifications for each measurement version. Unstandardized estimates are reported for the indirect associations, interaction coefficients, conditional indirect associations, and indices of moderated mediation. Values in brackets represent 95% bootstrap confidence intervals based on 5,000 bootstrap samples; intervals excluding zero indicate statistical significance. In Model 4, biospheric and hedonic values were included in the relevant regression equations to estimate each association while accounting for the other value. Biospheric value was included as a covariate in Models 1 and 7.

S4.2. Sensitivity Analysis Excluding Respondents Reporting "Never"

To assess the robustness of the findings, sensitivity analyses were conducted after excluding respondents who selected "Never" in the PBMA consumption-frequency question. Among the 288 respondents included in the main analyses, 41 selected this response category, leaving 247 respondents for the sensitivity analyses. The indirect-effect, moderation, and exploratory conditional indirect-effect analyses were repeated using the reduced sample. As shown in Table S6, the indirect associations of both biospheric and hedonic values with attitude through normative goal remained significant. The interaction between hedonic value and interdependent self-construal also remained significant, as did the index of moderated mediation. These results suggest that the main findings were generally consistent after excluding respondents who reported "Never" in the consumption-frequency question.

Table S6. Comparison of main analysis results before and after excluding respondents who selected "never" for PBMA consumption frequency.

Path / Statistic	Primary analytic sample (N = 288)	Respondents selecting "Never" excluded (N = 247)
	Indirect associations (Model 4)	
BV → NG → ATT	.333 [.226, .451]	.342 [.231, .474]
HV → NG → ATT	.120 [.029, .210]	.144 [.045, .246]
	Moderation results (Model 1)	
HV × Inter-SC (B)	.262	.255
p	.003	.009
ΔR^2	.023	.020
	Conditional indirect associations (Model 7)	
	HV → NG → ATT	
Low Inter-SC (−1 SD)	.031 [−.077, .131]	.037 [−.093, .150]
Mean Inter-SC	.161 [.058, .275]	.165 [.056, .283]
High Inter-SC (+1 SD)	.290 [.142, .473]	.293 [.131, .494]
Index of moderated mediation	.161 [.070, .284]	.157 [.049, .309]

Note. BV = biospheric value; HV = hedonic value; NG = normative goal; ATT = attitude toward PBMAs; Inter-SC = interdependent self-construal. Unstandardized estimates are reported. Brackets indicate 95% bootstrap confidence intervals for indirect and conditional indirect associations based on 5,000 bootstrap samples. For Model 4, biospheric and hedonic values were included in the relevant regression equations so that the association of each value was estimated while accounting for the other. BV was included as a covariate in Models 1 and 7. The same model specifications were applied to both samples.

S5. Ceiling Effect Assessment and Robustness Checks of the Moderation Analysis

Given the relatively high mean score for hedonic value (M = 6.030, SD = .833), additional analyses were conducted to assess potential ceiling effects and the robustness of the moderation results. Of the 288 respondents, 81 (28.1%) had the maximum composite hedonic value score of 7.00. Hedonic value scores ranged from 3.33 to 7.00, with a skewness of −.539, indicating a concentration of responses toward the upper end of the scale and suggesting a potential ceiling effect.

To assess the robustness of the moderation results, PROCESS Model 1 was re-estimated using HC3 heteroscedasticity-consistent standard errors, controlling for biospheric value. The interaction between hedonic value and interdependent self-construal remained statistically significant (B = .262, HC3 SE = .087, p = .003, 95% CI [.090, .434]).

Influence diagnostics identified 13 observations (4.5%) exceeding the exploratory Cook's distance threshold of 4/N (.0139). A sensitivity analysis excluding these observations (N = 275) showed that the interaction remained statistically significant (B = .422, HC3 SE = .076, p < .001, 95% CI [.272, .573]; Table S7). Although the magnitude of the interaction coefficient increased after exclusion, its positive direction and statistical significance were maintained. All 288 observations were retained in the primary analysis.

The interaction pattern was further illustrated using predicted normative goal scores at low (−1 SD), mean, and high (+1 SD) levels of interdependent self-construal (Figure S2).

Table S7. Robustness checks for the moderation analysis of interdependent self-construal in the association between hedonic value and normative goal.

Statistic	Primary analytic sample (N = 288)	Excluding influential observations (N = 275)
Interaction coefficient (B)	.262	.422
HC3 robust SE	.087	.076
t-value	3.004	5.523
p-value	.003	<.001
95% CI	[.090, .434]	[.272, .573]
R^2	.293	.348
ΔR^2	.023	.053
F(HC3) for interaction	9.025	30.498

Note. HV = hedonic value; NG = normative goal; Inter-SC = interdependent self-construal; BV = biospheric value; CI = confidence interval; SE = standard error. PROCESS Model 1 controlled for BV and used HC3 robust standard errors. Thirteen observations with Cook's distance > 4/N (.0139) were excluded only from the sensitivity analysis.

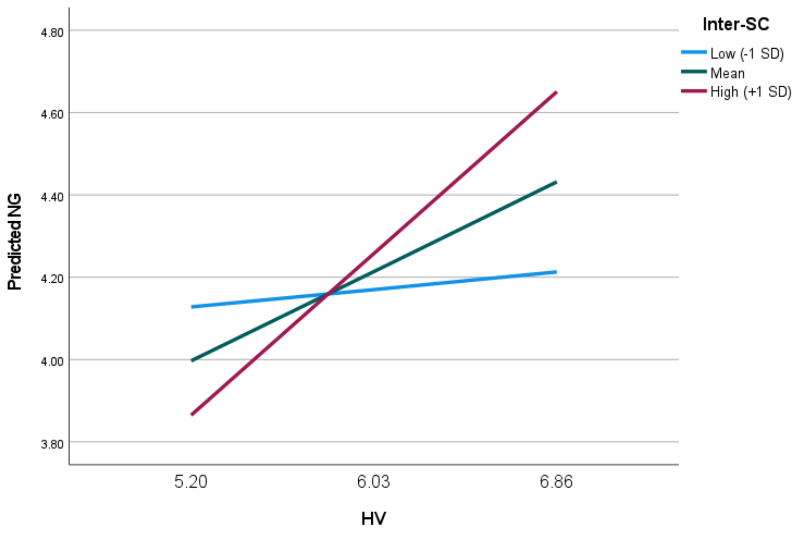

Figure S2. Interaction between hedonic value and interdependent self-construal in predicting normative goals.

Note. Lines represent predicted normative goal scores at low (−1 SD), mean, and high (+1 SD) levels of interdependent self-construal, controlling for biospheric value (N = 288).
